# Assessment of serum, dietary zinc levels, and other risk factors during the third trimester among pregnant women with and without pregnancy-induced hypertension: a case-control study

**DOI:** 10.3389/fnut.2023.1155529

**Published:** 2023-06-05

**Authors:** Abdel Hamid El Bilbeisi, Sahar M. Abo Khosa, Mahmoud H. Taleb, Amany M. El Afifi

**Affiliations:** ^1^Department of Food Science and Human Nutrition, College of Applied and Health Sciences, A’Sharqiyah University, Ibra, Oman; ^2^Department of Clinical Nutrition, Al-Azhar University – Gaza, Gaza, Palestine; ^3^Faculty of Pharmacy, Al-Azhar University – Gaza, Gaza, Palestine

**Keywords:** assessment, Palestine, pregnancy-induced hypertension, pregnant women, serum zinc level

## Abstract

**Background:**

This study assessed serum, dietary zinc levels, and other risk factors during the third trimester among pregnant women with and without pregnancy-induced hypertension (PIH).

**Methods:**

This case-control study was conducted in 2022, in the three main Obstetrics and Gynecology departments in Gaza Strip, Palestine. One hundred sixty pregnant women, during the third trimester, aged ≥20 years, were selected using a convenient sampling method. Data were obtained using an interview-based questionnaire, food frequency questionnaire, anthropometric measures, and biochemical tests. Statistical analysis was performed using SPSS version 24.

**Results:**

The participants’ mean age was 30.7 ± 5.6 years. A total of 47 (58.8%) of cases and 6 (7.5%) of controls were insufficiently active; and the mean of blood pressure (mmHg) was 133.3 ± 11.9/85.11 ± 10.0 for cases and 112.8 ± 9.5/68.02 ± 7.2 for controls with significant differences between the two groups (*P* = <0.005). The mean serum zinc level (μg/dl) was 67.15 ± 16.5 for cases and 68.45 ± 18.0 for controls without significant differences between the two groups (*P* = 0.636). For newborns, the mean birth weight (g) was 2,904.6 ± 486 for cases, and 3,128.3 ± 501 for controls, and the mean Apgar score was 8.03 ± 0.62 for cases and 8.30 ± 1.17 for controls, with significant differences between the two groups (*P* = <0.005). Furthermore, 43 (53.8%) of cases have family history of hypertension; 5 (6.2%) were primiparous; 19 (23.8%) have previous caesarian section; 33 (41.2%) have history of preeclampsia; and 62 (77.5%) have edema, with significant differences between the two groups (*P* = <0.005). Additionally, the total zinc dietary daily intake (mg/day) was 4.15 ± 2.10 for cases and 4.88 ± 3.02 for controls, with significant differences between the two groups (*P* = 0.041). After adjustment for confounding variables, participants in the case group have higher odds of having low total zinc dietary intake compared to those in the control group [OR = 1.185, 95% CI = (1.016–1.382), *P* = 0.030].

**Conclusion:**

The current study showed the main risk factors of PIH among pregnant women in the Gaza Strip, Palestine. Furthermore, low maternal dietary zinc intake was associated with a high level of PIH. Moreover, having PIH could increase the risk of low birth weight and low Apgar scores. Therefore, reducing the main risk factors of PIH could reduce the adverse effect on both mother and birth outcomes.

## Introduction

Zinc is an essential trace mineral element vital for many physiological functions and plays an important role in growth, reproduction, and the immune system (1). Adequate zinc stores in the body are extremely important during periods of pregnancy. However, zinc deficiency is common in developing countries, and low maternal serum zinc concentrations have previously been associated with pregnancy complications (2). Zinc deficiency during pregnancy adversely affects both the mother and fetus and subsequent birth outcomes. Major problems associated with zinc deficiency include growth retardation, delayed immune system development, cognitive impairment, impaired glucose tolerance, low birth weight, congenital malformations, pregnancy-induced hypertension (PIH), increased risk of abortion, miscarriage, stillbirths, preterm labor, postpartum hemorrhage, and prolonged labor (3).

Previous studies have provided evidence that zinc deficiency is a worldwide public health problem, especially in developing countries, including Palestine (4, 5). The World Health Organization (WHO) reported that the estimated prevalence of zinc deficiency ranges from 4 to 73% across various regions of the world’s population (6). In developing countries, zinc deficiency is 1 of the 10 significant factors contributing to the burden of disease (7). Zinc deficiency is predicted to be responsible for 1% of all deaths globally and 4.4% in children aged 6 months to 5 years (8). Additionally, the WHO prioritized minimizing zinc deficiency in developing countries to eradicate extreme poverty and hunger (9).

Diet is the main factor that determines zinc status (2). In the United States and Australia, an additional 2–4 mg of zinc per day is recommended for pregnant women compared to non-pregnant women (10, 11). It is widely acknowledged that many pregnant women do not meet this recommendation, particularly in developing countries with plant-based diets (12). Adequate maternal nutrition, particularly before and during pregnancy, is imperative to the mother’s and child’s health (13). Poor nutrition in pregnancy may lead to inappropriate nutrient partitioning between the mother and fetus, which can be deleterious to the health of both (14).

In fact, data on the prevalence and determinants of zinc deficiency among pregnant women are scanty and inconclusive (15). In addition, to the best of our knowledge, no baseline information exists about zinc levels and PIH among pregnant women in Gaza Strip, Palestine. Therefore, the aim of the current study was to assess the serum, dietary zinc levels, and other risk factors during the third trimester among pregnant women with and without PIH in Gaza Strip, Palestine, to determine whether maternal serum zinc or dietary zinc intake are important factors associated with PIH. Understanding this association may help improve maternal and fetal outcomes and reduce possible complications. This is the first study, which examined this association among pregnant women in Gaza Strip, Palestine, and the results of this study will provide baseline information about zinc levels and the main risk factors of PIH during the third trimester among pregnant women in Gaza Strip, Palestine.

## Materials and methods

### Study design

This case-control study was conducted among pregnant women attended the main governmental hospitals in Gaza Strip, Palestine.

### Study setting and period

This study was conducted in the year 2022 in the three main governmental hospitals (Al Shifa Medical Complex, Al-Aqsa Martyrs Hospital, and Mubarak Hospital) of the Palestinian Ministry of Health (MOH), which are considered the main governmental hospitals providing the Obstetrics and Gynecology services to the Palestinians pregnant woman.

### Study population

All pregnant women with and without PIH during the third trimester who attended the main governmental hospitals in Gaza Strip, Palestine, during the study period. The study protocol was approved by the Palestinian Health Research Council (PHRC/HC/116/22). The study was conducted according to the tenets of the Helsinki Declaration. Written informed consent was obtained from each participant. The inclusion criteria include pregnant women aged ≥20 years, women during the third trimester, women who were attending the Obstetrics and Gynecology Departments of Palestinian MOH during the study period and lived in the Gaza Strip for at least 3 years. The inclusion criteria for the cases included pregnant women diagnosed with PIH, and for the controls, we included pregnant women without PIH. On the contrary, pregnant women who were previously diagnosed with chronic hypertension (HTN) or other types of serious illness such as cancer, thyroid diseases, acute myocardial infarction, or end-stage kidney disease; pregnant women taking drugs affecting zinc balance; and pregnant women with a previous history of (antiphospholipid syndrome, ulcerative colitis, Crohn’s disease, short bowel syndrome, chronic diarrhea, chronic liver or kidney diseases, and sickle cell anemia) were also excluded.

### Sample size and sampling technique

The number of pregnant women who attended the governmental hospitals in the Gaza Strip in 2017 was 17.226. In addition, the number of high-risk pregnancies was 5,278 cases, according to a recent report from the Palestinian MOH in 2017 (16). The representative sample size in the current study was 160 pregnant women, which was calculated using the Charan and Biswas formula (17). Pregnant women during the third trimester, aged ≥20 years (80 cases and 80 controls) were selected by a convenient sampling method according to the availability of subjects who fit eligibility criteria during the data collocation period. Additionally, the study sample was distributed based on the population density on the three leading governmental hospitals in Gaza Strip, Palestine.

### Data collection

#### Interview-based questionnaire

Data regarding the characteristics of the study participants, demographic and socioeconomic, medical and gestational history, and diet compliance variables were obtained with an interview-based questionnaire. Additionally, reports and all relevant documentation, including the participant’s medical records, were checked.

### Anthropometric measurements

For pregnant women, height (cm) and weight (kg) were measured on admission using standard methods (18). Furthermore, for the newborns, length (cm), weight (g), and head and chest circumference (cm) were measured after delivery using standard methods (19).

### Blood pressure measurements (mmHg) for pregnant women on admission

Blood pressure was measured from the left arm (mmHg) by mercury sphygmomanometer. Three readings on different days, while the participant seated, after relaxing for at least 15 min in a quiet environment, empty bladder, and the average of three measurements was recorded (20).

### Biochemical measurements

After 12 h fasting, venous blood samples were collected from all participants (case and control) on admission and at the beginning of the third trimester by well-trained and experienced nurses. Venous blood (5.0 ml) was drawn and was used for serum zinc level (μg/dl), hemoglobin level (g/dl), and fasting blood sugar (mg/dl). Mindray BS-300 and Mindray BA-88A chemistry analyzers were used for blood chemistry and serum zinc level analysis (21). In addition, serum zinc was analyzed by colorimetric method of Mindray BA-88A analyzer, using commercially available colorimetric determination kits of serum zinc. This is a direct colorimetric assay based on the 5-Br-PAPS method. The laboratory tests were analyzed in a private licensed laboratory.

### Clinical examination

All participants were examined by the physicians for signs and symptoms of any diseases, including PIH, and for signs and symptoms of zinc deficiency. In addition, the WHO criteria were used by the physicians for the diagnosis of PIH (22). In the current study, PIH is defined as systolic blood pressure >140 mmHg and diastolic blood pressure >90 mmHg. It is refers to one of four conditions: (a) pre-existing hypertension, (b) gestational hypertension and preeclampsia, (c) pre-existing hypertension plus superimposed gestational hypertension with proteinuria, and (d) unclassifiable hypertension (22–24).

### Apgar score

The Apgar score was used to assess the health status of the newborn. The Apgar score is a quick way for various members of the healthcare team, including midwives, nurses, or physicians, to evaluate the health of all newborns at 1 and 5 min after birth and in response to resuscitation (25).

### Dietary assessment

In the current study, a validated Palestinian Arabic version semi-quantitative food frequency questionnaire (FFQ) was used to assess the dietary zinc intake of the pregnant women (26). The FFQ is relatively easy and inexpensive to administer and can be used to measure dietary intake over a prolonged time period, in addition the semi-quantitative FFQ is adequate to identify deficiency of zinc and other trace elements (20). The FFQ in our study contains a list of 98 food items; it was developed and validated among the Palestinian population in 2014 (26). All participants were asked to estimate the number of times per day, week, or month they consumed these particular food products and the amount usually eaten per food item by making comparisons with the specified reference portion. Common household measures, including measuring cups, spoons, and a ruler were shown to assist the participants in the estimation process. Additionally, dietary intakes were converted into grams per day. The net amount of dietary zinc intake (mg/day) consumed by the study participants was calculated based on the USDA Food Composition Database (27).

### Assessment of physical activity levels

Data on physical activity was obtained using the international physical activity questionnaire (IPAQ) short version. According to the IPAQ scoring protocol, the participants were classified based on their weekly energy expenditure as follows: insufficiently active: ≤600 MET/week; sufficiently active: 601–1,500 MET/week; and very active: ≥1,500 MET/week (28).

### Pilot study

Pilot study was carried out on 20 participants; the questionnaire and data collection process were modified according to the result of the pilot study. The data was collected by five qualified data collectors (two nurses and three nutritionists).

### Statistical analysis

Statistical analysis was performed using SPSS version 24. Dietary intakes were converted into grams per day. The net amount of zinc dietary intake (mg/day) consumed by the study participants were calculated based on the USDA Food Composition Database (27). In addition, data are expressed as means ± standard deviation (SD) for continuous variables and as percentage for categorical variables. The Chi-square test and Fisher’s exact test were used to examine the significant differences in the prevalence of different categorical variables. The differences between mean were tested by independent samples *t*-test. *P*-value less than 0.05 was considered as statistically significant.

## Results

The current study was conducted in the three main governmental hospitals of the Palestinian MOH. The results demonstrated that the majority of the study participants were from Al Shifa Medical Complex and Mubarak Hospital. The mean of age for the study participants was 30.7 ± 5.6 years without significant differences between the cases and control groups (*P* = 0.067). Concerning physical activity levels, a large percentage of the cases were insufficiently active, while the majority of controls were very active, with significant differences found between the case and control group (*P* = 0.001). In addition, the mean blood pressure for women in the cases group was slightly high compared to women in controls group, but still within the normal levels, with significant differences between the two groups (*P* = < 0.005). Moreover, the mean serum zinc and hemoglobin levels was slightly low for women in the cases group compared with women in the control group; while the mean of fasting plasma glucose was slightly high compared to women in controls group, without significant differences between the two groups (*P* > 0.005 for all), as shown in Table 1.

**TABLE 1:** Characteristics of the study participants.

Variables	Total (*n* = 160)	Case (*n* = 80)	Control (*n* = 80)	*P*- value
	**No. (%)**	**No. (%)**	**No. (%)**	
**Hospital name**				
Al Shifa Medical Complex	60.0 (37.5)	30.0 (37.5)	30.0 (37.5)	–
Al-Aqsa Martyrs Hospital	40.0 (25.0)	20.0 (25.0)	20.0 (25.0)	
Mubarak Hospital	60.0 (37.5)	30.0 (37.5)	30.0 (37.5)	
**Age (years)**				
Mean ± SD	30.7 ± 5.6	31.5 ± 6.2	29.9 ± 4.9	0.067
**Education level**				
Low (primary and preparatory)	87.0 (54.3)	40.0 (50.0)	47.0 (58.7)	0.599
High (secondary and university)	73.0 (45.7)	40.0 (50.0)	33.0 (41.2)	
**Work status**				
Has work	10.0 (6.2)	7.0 (8.8)	3.0 (3.8)	0.328
Do not have work	150 (93.8)	73.0 (91.2)	77.0 (96.2)	
**Number of family members**				
<5	97.0 (60.6)	43.0 (53.8)	54.0 (67.5)	0.164
5–10	59.0 (36.9)	34.0 (42.5)	25.0 (31.2)	
>10	4.0 (2.5)	3.0 (3.8)	1.0 (1.2)	
**Monthly income (NIS)**				
<1,000 (NIS)	115 (71.9)	51.0 (63.8)	64.0 (80.0)	0.067
1,000–2,000 (NIS)	43.0 (26.9)	28.0 (35.0)	15.0 (18.8)	
2,001–3,000 (NIS)	2.0 (1.2)	1.0 (1.2)	1.0 (1.2)	
**Enough income**				
Yes	66.0 (41.2)	38.0 (47.5)	28.0 (35.0)	0.148
No	94.0 (58.8)	42.0 (52.5)	52.0 (65.0)	
**Physical activity levels (MET/week)**				
Insufficiently active (≤600)	53.0 (33.1)	47.0 (58.8)	6.0 (7.5)	0.001
Sufficiently active (601–1,500)	44.0 (27.5)	21.0 (26.2)	23.0 (28.8)	
Very active (≥1,500)	63.0 (39.4)	12.0 (15.0)	51.0 (63.8)	
**Systolic blood pressure (mmHg)**				
Mean ± SD	123.1 ± 14.8	133.3 ± 11.9	112.8 ± 9.5	0.001
**Diastolic blood pressure (mmHg)**				
Mean ± SD	76.56 ± 12.2	85.11 ± 10.0	68.02 ± 7.2	0.001
**Serum zinc level (μg/dl)**				
Mean ± SD	67.80 ± 17.2	67.15 ± 16.5	68.45 ± 18.0	0.636
**Fasting plasma glucose (mg/dl)**				
Mean ± SD	83.83 ± 15.5	84.66 ± 18.0	83.0 ± 12.61	0.273
**Hemoglobin level (g/dl)**				
Mean ± SD	10.90 ± 1.19	10.93 ± 1.33	10.88 ± 1.05	0.065

Data are expressed as means ± SD for continuous variables and as percentage for categorical variables. The differences between means were tested by using an independent sample *t*-test. The Chi-square test was used to examine differences in the prevalence of different categorical variable. *P*-value less than 0.05 was considered as statistically significant. SD, stander deviation; NIS, New Israeli Shekel; MET/week, metabolic equivalents per week.

Table 2 presented the anthropometric measures for the mothers and for the newborns. For mothers, the results showed that the mean BMI (kg/m^2^) was slightly high for women in cases group compared with women in the controls group, without significant differences between the two groups (*P* > 0.005). For newborns, the results revealed that the mean birth weight (g) and the mean of Apgar score were low in the cases group compared with the controls group, with significant differences between the two groups (*P* = 0.005 and 0.001, respectively).

**TABLE 2:** Anthropometric measurements for the study participants, newborns, and the results of Apgar score.

Variables	Total (*n* = 160)	Case (*n* = 80)	Control (*n* = 80)	*P*-value
	**No. (%)**	**No. (%)**	**No. (%)**	
**1. Anthropometric measurements for pregnant women on admission**				
**Height (cm)**				
Mean ± SD	161.4 ± 6.11	161.38 ± 6.68	161.60 ± 5.52	0.827
**Weight (kg)**				
Mean ± SD	81.16 ± 13.8	82.86 ± 14.8	79.47 ± 12.6	0.122
**Body mass index (BMI): kg/m^2^**				
Mean ± SD	31.16 ± 5.37	31.88 ± 5.85	30.44 ± 4.77	0.091
**Classification of BMI (kg/m^2^)**				
Underweight (BMI <18.5 kg/m^2^)	1.0 (0.6)	0.0 (0.0)	1.0 (1.2)	0.268
Normal weight (BMI 18.5–24.9 kg/m^2^)	15.0 (9.4)	5.0 (6.2)	10.0 (12.5)	
Overweight (BMI 25–29.9 kg/m^2^)	52.0 (32.5)	30.0 (37.5)	22.0 (27.5)	
Obesity (BMI ≥30 kg/m^2^)	92.0 (57.5)	45.0 (56.2)	47.0 (58.8)	
**2. Anthropometric measurements for newborns after delivery**				
**Length (cm)**				
Mean ± SD	47.63 ± 3.15	47.30 ± 2.96	47.96 ± 3.32	0.185
**Weight (g)**				
Mean ± SD	3016.5 ± 505	2904.6 ± 486	3128.3 ± 501	0.005
**Head circumference (cm)**				
Mean ± SD	34.84 ± 1.58	34.94 ± 1.53	34.73 ± 1.64	0.413
**Chest circumference (cm)**				
Mean ± SD	33.20 ± 2.96	33.23 ± 1.45	33.17 ± 3.94	0.090
**Apgar score/10**				
Mean ± SD	8.16 ± 0.94	8.03 ± 0.62	8.30 ± 1.17	0.001

Data are expressed as means ± SD for continuous variables. The differences between means were tested by using independent sample *t*-test. *P*-value less than 0.05 was considered as statistically significant. SD, stander deviation.

Table 3 presents selected factors that may contribute to the development of PIH. The results showed that a large percentage of the cases have a family history of HTN compared with the controls, with a statistically significant difference between the two groups (*P* = 0.028). In addition, women in the cases group have more family history of DM compared with the controls, without significant differences between the two groups (*P* = 0.867). The results also revealed that women in the cases group have more history of preeclampsia and complained of edema more than women in the controls group, with significant differences between the two groups (*P* < 0.005 for all).

**TABLE 3:** Medical and gestational history variables of the study participants.

Variables	Total (*n* = 160)	Case (*n* = 80)	Control (*n* = 80)	*P*-value
	**No. (%)**	**No. (%)**	**No. (%)**	
**Family history of diabetes mellitus**				
Yes	54.0 (33.8)	28.0 (35.0)	26.0 (32.5)	0.867
No	106 (66.2)	52.0 (65.0)	54.0 (67.5)	
**Family history of hypertension**				
Yes	73.0 (45.6)	43.0 (53.8)	30.0 (37.5)	0.028
No	87.0 (54.4)	37.0 (46.2)	50.0 (62.5)	
**Do you have lipid abnormality or take medications for lipid abnormality?**				
Yes	6.0 (3.8)	4.0 (5.0)	2.0 (2.5)	0.341
No	154 (96.2)	76.0 (95.0)	78.0 (97.5)	
**Do you have diabetes mellitus or use a specific treatment of previously diagnosed diabetes mellitus?**				
Yes	8.0 (5.0)	5.0 (6.2)	3.0 (3.8)	0.360
No	152 (95.0)	75.0 (93.8)	77.0 (96.2)	
**Do you expose to second-hand smoke?**				
Yes	65.0 (40.6)	37.0 (46.2)	28.0 (35.0)	0.198
No	95.0 (59.4)	43.0 (53.8)	52.0 (65.0)	
**History of smoking or alcohol intake**				
No	160 (100)	80.0 (100)	80.0 (100)	–
**Mode of conception**				
Natural conceived	150 (93.8)	75.0 (93.8)	75.0 (93.8)	0.254
*In vitro* fertilization-embryo transfer	10.0 (6.2)	5.0 (6.2)	5.0 (6.2)	
**Number of pregnancy**				
Nulliparous	48.0 (30.0)	29.0 (36.2)	19.0 (23.8)	0.009
Primiparous	23.0 (14.4)	5.0 (6.2)	18.0 (22.5)	
Multiparous	89.0 (55.6)	46.0 (57.5)	43.0 (53.8)	
**History of abortion**				
Yes	44.0 (27.5)	25.0 (31.2)	19.0 (23.8)	0.288
No	116 (72.5)	55.0 (68.8)	61.0 (76.2)	
**History of gestational diabetes mellitus**				
Yes	12.0 (7.5)	9.0 (11.2)	3.0 (3.8)	0.072
No	148 (92.5)	71.0 (88.8)	77.0 (96.2)	
**History of big baby**				
Yes	20.0 (12.5)	12.0 (15.0)	8.0 (10.0)	0.339
No	140 (87.5)	68.0 (85.0)	72.0 (90.0)	
**History of previous caesarian section**				
Yes	55.0 (34.4)	19.0 (23.8)	36.0 (45.0)	0.004
No	105 (65.6)	61.0 (76.2)	44.0 (55.0)	
**History of hypertensive disorders during pregnancy (preeclampsia)**				
Yes	35.0 (21.9)	33.0 (41.2)	2.0 (2.5)	0.001
No	125 (78.1)	47.0 (58.8)	78.0 (97.5)	
**History of anemia**				
Yes	33.0 (20.6)	20.0 (25.0)	13.0 (16.2)	0.171
No	127 (79.4)	60.0 (75.0)	67.0 (83.8)	
**Complain of edema**				
Yes	83.0 (51.9)	62.0 (77.5)	21.0 (26.2)	0.001
No	77.0 (48.1)	18.0 (22.5)	59.0 (73.8)	

Data are expressed as percentage for categorical variables. The Chi-square test and Fisher’s exact test were used to examine differences in the prevalence of different categorical variables. *P*-value less than 0.05 was considered as statistically significant.

Table 4 showed that a large percentage of cases have a meal plan and have three meals per day, without significant differences between the two groups (*P* < 0.005). In addition, women in the cases group using dietary supplements, including multivitamins, received large amounts of iron supplementation, and used zinc supplementation more than women in the controls group, without significant differences between the two groups (*P* = >0.05 for all).

**TABLE 4:** Diet compliance variables of the study participants.

Variables	Total (*n* = 160)	Case (*n* = 80)	Control (*n* = 80)	*P*-value
	**No. (%)**	**No. (%)**	**No. (%)**	
**Do you have a meal plan?**				
Yes	39.0 (24.4)	27.0 (33.8)	12.0 (15.0)	0.005
No	121 (75.6)	53.0 (66.2)	68.0 (85.0)	
**Numbers of meals per day**				
Less than three	48.0 (30.0)	23.0 (28.8)	25.0 (31.2)	0.645
Three meals	95.0 (59.4)	50.0 (62.5)	45.0 (56.2)	
More than three	17.0 (10.6)	7.0 (8.8)	10.0 (12.5)	
**Dietary supplement use (including multivitamins)**				
Yes	113 (70.6)	61.0 (76.2)	52.0 (65.0)	0.082
No	47.0 (29.4)	19.0 (23.8)	28.0 (35.0)	
**Do you receive large amounts of iron supplementation?**				
Yes	132 (82.5)	69.0 (86.2)	63.0 (78.8)	0.149
No	28.0 (17.5)	11.0 (13.8)	17.0 (21.2)	
**Zinc supplementation**				
Yes	8.0 (5.0)	5.0 (6.2)	3.0 (3.8)	0.360
No	152 (95.0)	75.0 (93.8)	77.0 (96.2)	

Data are expressed as percentage for categorical variables. The Chi-square test was used to examine differences in the prevalence of different categorical variable. *P*-value less than 0.05 was considered as statistically significant.

Table 5 presents the consumption patterns of dietary zinc intake (mg/day). The total dietary zinc daily intake (mg/day) were slightly low for women in the cases group compared with women in the control group, with significant differences between the two groups (*P* = 0.041).

**TABLE 5:** Consumption pattern of zinc dietary intake (mg/day) of the study participants.

Food items	Total (*n* = 160)	Case (*n* = 80)	Control (*n* = 80)	*P*-value
	**No. (%)**	**No. (%)**	**No. (%)**	
**1. Beef meat (zinc 3.24 mg/100 g)**				
Mean ± SD	0.326 ± 0.811	0.335 ± 0.839	0.318 ± 0.788	0.893
**2. Poultry (zinc 1.68 mg/100 g)**				
Mean ± SD	0.252 ± 0.220	0.219 ± 0.133	0.285 ± 0.278	0.004
**3. Beef liver (zinc 8.40 mg/100 g)**				
Mean ± SD	0.465 ± 1.44	0.480 ± 0.650	0.450 ± 1.95	0.449
**4. Fresh and canned fish (zinc 0.51 mg/100 g)**				
Mean ± SD	0.034 ± 0.075	0.037 ± 0.089	0.031 ± 0.313	0.453
**5. Shellfishes (zinc 1.87 mg/100 g)**				
Mean ± SD	0.043 ± 0.174	0.063 ± 0.229	0.023 ± 0.087	0.008
**6. Eggs (zinc 0.88 mg/1 egg: 50 g)**				
Mean ± SD	0.428 ± 0.434	0.364 ± 0.411	0.491 ± 0.450	0.218
**7. Milk, dairy beverages (zinc 1.05 mg/300 g: large glass)**				
Mean ± SD	0.540 ± 0.593	0.519 ± 0.559	0.560 ± 0.628	0.286
**8. Dark breads, breads with grains (zinc 0.66 mg/30 g: slice)**				
Mean ± SD	0.153 ± 0.330	0.166 ± 0.333	0.141 ± 0.330	0.361
**9. Pasta (zinc 0.89 mg/1 glass: cooked)**				
Mean ± SD	0.152 ± 0.293	0.123 ± 0.169	0.181 ± 0.378	0.018
**10. Rice (zinc 1.21 mg/1 glass: cooked)**				
Mean ± SD	0.366 ± 0.428	0.328 ± 0.394	0.404 ± 0.458	0.381
**11. White bread, wheat bread, baguette, rolls, toast bread (zinc 0.31 mg/30 g: slice, half of a roll)**				
Mean ± SD	0.366 ± 0.186	0.332 ± 0.159	0.400 ± 0.205	0.001
**12. Fruits (zinc 0.17 mg/100 g: half of a glass)**				
Mean ± SD	0.111 ± 0.097	0.100 ± 0.082	0.122 ± 0.110	0.010
**13. Legumes (zinc 1.61 mg/100 g)**				
Mean ± SD	0.497 ± 0.718	0.454 ± 0.606	0.540 ± 0.817	0.221
**14. Other vegetables (zinc 0.45 mg/100 g: 1 glass of leafy vegetables, half of a glass of the other**				
Mean ± SD	0.505 ± 0.296	0.462 ± 0.258	0.548 ± 0.325	0.001
**15. Pumpkin and flax seeds (zinc 2.29 mg/30 g: handful)**				
Mean ± SD	0.079 ± 0.417	0.069 ± 0.278	0.090 ± 0.523	0.495
**16. Other nuts and seeds (zinc 0.87 mg/30 g: handful)**				
Mean ± SD	0.246 ± 0.407	0.197 ± 0.331	0.294 ± 0.467	0.026
Total zinc dietary intake (mg/day)	4.56 ± 2.62	4.15 ± 2.10	4.88 ± 3.02	0.041

Data are expressed as means ± SD for continuous variables. The differences between means were tested by using an independent sample *t*-test. *P*-value less than 0.05 was considered as statistically significant. SD, stander deviation.

Finally, Table 6 presented the odd ratio (OR) and confidence interval (CI) for serum zinc level (μg/dl) and total dietary zinc intake (mg/day) of the case and control group. After adjustment for confounding variables, participants in the case group have higher odds of having low total zinc dietary intake, compared to those in the control group [OR = 1.185, 95% CI = (1.016–1.382), *P* = 0.030].

**TABLE 6:** Odd ratio and confidence interval for the serum zinc level (μg/dl) and total dietary zinc intake (mg/day) of the case and control group.

Variables	Case (*n* = 80)	Control (*n* = 80)	*P*-value	OR (95% CI)
	**Mean ± SD**	**Mean ± SD**		
Serum zinc level (μg/dl)	67.15 ± 16.5	68.45 ± 18.0	0.633	1.004 (0.986–1.023)
Adjusted*			0.280	1.012 (0.990–1.035)
Total zinc dietary intake (mg/day)	4.15 ± 2.10	4.88 ± 3.02	0.134	1.102 (0.970–1.252)
Adjusted*			0.030	1.185 (1.016–1.382)

The OR and CI for the serum zinc level (μg/dl) and total dietary zinc intake (mg/day) of the case and control group were tested by binary logistic regression.

*Adjusted for physical activity levels (MET/week), systolic and diastolic blood pressure (mmHg), weight (g), results of Apgar score, family history of hypertension, number of pregnancies, history of previous caesarian section, history of hypertensive disorders during pregnancy (preeclampsia), complain of edema, and have a meal plan.

*P*-value less than 0.05 was considered as statistically significant.

OR, odds ratio; CI, confidence interval.

## Discussion

Zinc deficiency is common in developing countries, and low maternal serum zinc concentrations have previously been associated with pregnancy complications (2). Zinc deficiency during pregnancy has an adverse effect on both the mother and fetus and on subsequent birth outcomes. Previous studies have provided evidence that zinc deficiency is a worldwide public health problem, especially in developing countries (4, 5). In the current study, no statistically significant associations were found concerning socioeconomic variables with the risk of PIH between the case and control group. Owiredu et al. (29) also showed that socioeconomic status was not directly associated with PIH. However, low socioeconomic factors may act as risk factors for PIH (30). Low socioeconomic factors could be associated with nutritional issues, reduced antenatal care and unsanitary hygienic conditions (31). A study in Australia found that working women compared to non-working ones, had a higher risk of developing pre-eclampsia and eclampsia; this may be related to the stress that women get during work (32). Several previous studies showed significant associations between maternal education and income level with PIH (30, 33).

Two of previous studies showed that aerobic exercise for about 30–60 min for at least twice per week during pregnancy is associated with a reduced risk of gestational hypertensive disorders, PIH, and cesarean delivery (34, 35). Our results support these findings of these studies.

The International Zinc Nutrition Consultative Group defined zinc deficiency as blood zinc levels below 56 μg/dl in the first trimester or 50 μg/dl in the second or third trimesters (36). Tesfa et al. (37) showed that the mean serum zinc level was significantly reduced in women with PIH as compared to normotensive pregnant women. On the contrary, Lewandowska et al. (38) showed no association between serum zinc concentrations and PIH. The same results were obtained by Mistry et al. (39) and Ugwuja et al. (40). The results of the current study, indicated that all of the study participants have normal serum zinc level, fasting plasma glucose, and hemoglobin level during the study period, therefore, it is not yet clear whether zinc levels play a role in PIH.

van Middendorp et al. (41) showed that having family history of HTN had about five time’s greater odds of developing PIH than those who did not have family history of HTN. This is consistent with the results of our study. Umegbolu and Ogamba (42) reported that first pregnancy is a risk factor for PIH and its occurrence is more common in nulliparous than multiparous women. Howell (43) showed that in women with prior cesarean delivery, PIH was significantly higher. The results of our study support these findings. The etiology of PIH is not fully understood yet, among many factors, genetic, metabolic, immunological, and environmental factors are taken into consideration (44, 45). Further future studies with large sample size on the main risk factors for PIH are recommended.

Hromadnikova et al. (45) demonstrated that there was a significant association of PIH with low birth weight, and women who delivered low birth weight babies were five times more likely to have had PIH. Furthermore, high blood pressure during pregnancy can affect the development of the placenta, causing the nutrient and oxygen supply to the baby to be limited, which associated with a lower mean Apgar score (46). In agreement with these studies, our results demonstrated that, for newborns, the mean birth weight (g) and the mean Apgar score were lower in the cases compared to women in the control group.

Pregnant women need up to 2.6 mg of zinc per day during the second half of their pregnancy (47). The WHO recommends a zinc intake between 1.1 and 2.0 mg/day for pregnant mothers (48). However, studies have reported that people in low and middle-income nations tend to eat plant-based foods that are high in phytate, and starchy roots and tubers, which have a low zinc concentration, which could lead to zinc insufficiency (49, 50). We found a lower intake of zinc during pregnancy in our cohort, although this does not correlate with low serum zinc levels suggesting that the study participants have adequate total zinc intake during pregnancy. Hennigar et al. (51) showed that dietary zinc intake is not substantially correlated with serum zinc concentration, because many factors have been identified to affect serum zinc independent of an individual’s zinc status, such as meal consumption, time of day, inflammation, and infection. However, as zinc intake appears to correlate with the frequency of PIH a changing diet might be warranted. Increasing the intake of animal-source foods, cutting out or cutting back on coffee, and taking zinc supplements could improve (52).

Previous studies showed that inadequate nutrition at home could be attributed to a number of factors, including lack of variety in the diet, low consumption of animal products, socioeconomic status, maternal education, pregnancy, hemoglobin concentration, maternal workload, chronic illness, vegetarianism, and a lack of antenatal care (53, 54) can contribute to inefficient zinc intake. Further research on the relationship between serum and dietary zinc levels during the third trimester among pregnant women with and without PIH and pregnancy outcomes needs more studies in the future.

### Strengths and limitations

The main strength of this study is that the results will provide baseline information about zinc levels and PIH during the third trimester among pregnant women in Gaza Strip, Palestine. The main limitations of this study are its non-probability sampling techniques, which limit the generalizability of the results. Furthermore, the possibility of recall bias and misreporting by using FFQ for maternal dietary zinc intake. Unfortunately, we did not record the gestational age at the time of blood collection nor at the time of delivery.

## Conclusion

The current study showed the main risk factors of PIH among pregnant women in the Gaza Strip, Palestine are family history of hypertension, primiparous, previous caesarian section, history of preeclampsia, complaints of edema, high maternal BMI, physical inactivity, and low maternal dietary zinc intake. Furthermore, low maternal dietary zinc intake was associated with high levels of PIH. Moreover, having PIH could increase the risk of low birth weight and low Apgar scores. Therefore, reducing the main risk factors of PIH could reduce the adverse effect on both mother and birth outcomes. Further future studies with large sample size are recommended.

## Data availability statement

The original contributions presented in this study are included in the article/supplementary material, further inquiries can be directed to the corresponding author.

## Ethics statement

The study protocol was approved by the Palestinian Health Research Council (Helsinki Ethical Committee of Research PHRC/HC/116/22). The patients/participants provided their written informed consent to participate in this study.

## Author contributions

SA and AME collected, analyzed, and interpreted the data and wrote the first draft of the manuscript. SA, AME, MT, and AHE contributed in the study design and the critical review of the manuscript. AHE contributed to the analysis and interpretation of data and the critical review of the manuscript. All authors contributed to the article and approved the submitted version.
